# Efficacy and safety of SOF/LDV in HCV-infected children and adolescents on hemodialysis: a prospective single-center observational study

**DOI:** 10.1007/s00467-025-07112-6

**Published:** 2026-03-09

**Authors:** Afaf Enayet, Hanaa El-Karaksy, Engy Mogahed, Noha Yasin, Haytham Ghita, Yasmin Ramadan

**Affiliations:** Pediatric Hepatology Unit and Pediatric Nephrology Unit, Pediatrics Department, Kasr AlAiny School of Medicine, Cairo University, Giza, Egypt

**Keywords:** Chronic kidney disease, CKD, DAAs, HCV, Hemodialysis, Pediatrics, Sofosbuvir/ledipasvir, SOF/LDV

## Abstract

**Background:**

To evaluate the efficacy and safety of sofosbuvir (SOF)-based direct-acting antivirals (DAAs) in HCV-infected pediatric chronic kidney disease (CKD) patients on maintenance hemodialysis (HD).

**Methods:**

This is a single-center, prospective single-arm observational exploratory study assessing the efficacy and safety of a 12-week course of sofosbuvir/ledipasvir (SOF/LDV) for the treatment of chronic HCV in Egyptian children and adolescents, 6–17 years old, who have CKD on regular HD. Potential adverse events (AE) were reported at baseline and during treatment administration using a detailed questionnaire. Sustained virological response (SVR12) was the primary study outcome.

**Results:**

The present study included 22 CKD patients on maintenance HD therapy. Their age ranged from 7.5 to 15.9 years with a median (IQR) of 12 (9.25–13.5) years. They were diagnosed with CKD stage 5 and scheduled for regular HD at a median (IQR) age of 6 (3.5–9) years (range 2–12 years). The median (IQR) baseline quantitative HCV RNA was 62,446 (6875–604,000) IU/L. During treatment, one patient experienced severe vomiting necessitating discontinuation of SOF/LDV. The remaining 21 patients completed the treatment course. The reported AE were all mild, and there were no reported serious adverse events. Nineteen out of 22 patients (86.4%) achieved SVR12.

**Conclusions:**

SOF/LDV was found to be effective in the treatment of HCV in children and adolescents with CKD on HD with SVR12 86.4%, with no serious adverse events recorded.

**Graphical abstract:**

**Figure:**
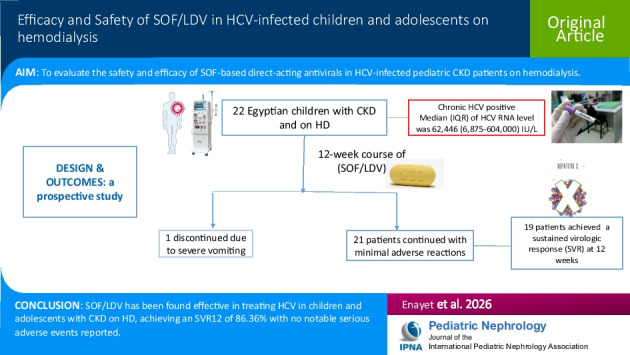
A higher resolution version of the Graphical abstract is available as Supplementary information

**Supplementary Information:**

The online version contains supplementary material available at 10.1007/s00467-025-07112-6.

## Introduction

Patients with chronic kidney disease (CKD) stage 5 on maintenance hemodialysis (HD) are known to have a high prevalence of hepatitis C virus (HCV) infection [1]. Moreover, HCV infection is incriminated as a risk factor for developing CKD, accelerating its progression, worsening the prognosis of HD therapy, and reducing the survival of kidney grafts [2]. Thus, HCV screening and anti-HCV therapy are highly recommended in such high-risk populations [3]. Interferon (IFN)-free direct-acting antiviral (DAA) therapies are the currently approved treatment strategy. Nevertheless, careful consideration should be warranted while administering DAA. Sofosbuvir (SOF), one of the most potent and widely used DAAs, is metabolized via the kidney. Also, drug–drug interactions (DDI) should be closely monitored, as patients on HD generally have complex prescriptions [2]. The US Food and Drug Administration (FDA) granted SOF-containing regimens approval for use in patients with estimated glomerular filtration rate (eGFR) < 30 mL/min/1.73 m^2^ including those on maintenance HD therapy [4]. Many studies have evaluated the efficacy and safety of DAA regimens in adult patients with CKD, including those on HD and after kidney transplantation [5]. To our knowledge, this is the first study to evaluate the efficacy and safety of the SOF-based DAA regimen in pediatric CKD patients on maintenance HD.

## Patients and methods

### Ethical considerations

The study protocol was reviewed and approved, ethically and scientifically, by the Cairo University Research Ethics Committee (REC: A-4–2021). Written consents were obtained from patients’ guardians as well as patients’ assents before drug administration, where risks and benefits were thoroughly discussed.

#### Study design

A single-center, prospective single-arm observational exploratory study was conducted to assess the efficacy and safety of a single oral, weight-based, daily dose of sofosbuvir/ledipasvir (SOF/LDV) for the treatment of chronic HCV in Egyptian children and adolescents, 6–17 years old, who have CKD on regular HD. Twenty-two patients were recruited from the Pediatric Nephrology and Hepatology Units, Cairo University Pediatric Hospital, Faculty of Medicine, Cairo University, with proven HCV infection with polymerase chain reaction (PCR) for at least 6 months before enrollment from May 2021 to September 2024. Genotyping was not done as it is well-known that the commonest genotype in Egypt (91%) is genotype 4 [6, 7]; moreover, most DAAs are pangenotypic.

#### Patient exclusion criteria

All patients eligible for treatment presented during the study period were enrolled; the following patients were excluded:

1. Patients with history of chronic hepatitis B virus (HBV) infection [positive test for hepatitis B surface antigen (HBsAg) or antibody to hepatitis B core antigen (HBcAb IgG)].
2. Patients with history of liver or any other organ transplant (including hematopoietic stem cell transplants).
3. Patients with history of current or known history of active or suspected malignancy.
4. Patients with a history of significant or unstable cardiac disease, uncontrolled cardiac arrhythmias, patients requiring any anti-arrhythmic drugs that have significant DDI with study drug, or pace-maker insertion.
5. Subjects on medications with significant DDI with the study drug.

#### Drug dosing

Patients received a 12-week course of SOF/LDV tablets as a single oral weight-based daily dose after the dialysis session. No dose adjustment was required as all patients were on regular HD [8]. Patients received weight-based dosing as follows: patients with weight 17  to < 35 kg: sofosbuvir 200 mg/ledipasvir 45 mg (half tablet), while patients weighing ≥ 35 kg: sofosbuvir 400 mg/ledipasvir 90 mg [9–11].

#### Methods

All enrolled subjects completed the following:

A Full history taking (primary kidney disease, duration of HD, duration of HCV infection, other hepatic symptoms), concomitant medications, physical examination.
B Laboratory tests including complete blood count (CBC), total and direct serum bilirubin, aspartate aminotransferase (AST), alanine aminotransferase (ALT), alkaline phosphatase (AP), gamma glutamyl transpeptidase (GGT), serum albumin, prothrombin time (PT) and international normalized ratio (INR), kidney function tests, and HCV viral load as illustrated in Table 1.
C Hepatitis B screening was done prior to treatment by HBsAg and HBcAb. Besides antibodies to HBsAg, i.e., anti-HBs, antibody titer was measured to assess the need for booster of hepatitis B vaccine.
D For cardiac assessment, all patients underwent 12-lead electrocardiogram (ECG), 24-h Holter ECG, in addition to echocardiographic study.
E Fibrosis assessment at baseline using FibroScan (FibroScan Echosens, Paris, France).
F Adverse events (AE) were reported at baseline and during treatment administration using a detailed questionnaire. AE was defined as any unfavorable or unintended sign, symptom, or disease temporally associated with the use of the drug, regardless of its causal relationship with the administrated drug. A serious adverse event (SAE) was defined as any AE that resulted in death, was life-threatening, required inpatient hospitalization or prolongation of existing hospitalization, resulted in persistent or significant disability/incapacity, or was a congenital anomaly/birth defect. The severity of AEs was graded from 1 (mild) to 5 (death) according to the Common Terminology Criteria for Adverse Events (CTCAE), version 5.0 [12].

In every visit, drug dosing, compliance, and tolerance were assessed throughout the study (Table 1).

**Table 1:** The fulfilled requirements at baseline and in the follow-up visits for all patients

	Baseline	Week 4	Week 12	Week 24
Physical examination	✓	✓	✓	✓
HCV RNA	✓	✓	✓	✓
CBC	✓	✓	✓	✓
Liver profile*	✓	✓	✓	✓
Renal function tests**	✓	✓	✓	✓
HBV screening	✓			
12-lead ECG***	✓	✓	✓	
24-h Holter ECG****	✓		✓	
Echocardiography	✓			
FibroScan	✓			
Adverse events	✓	✓	✓	✓
Drug monitoring	✓	✓	✓	✓

*Liver profile: total and direct serum bilirubin, aspartate aminotransferase (AST), alanine aminotransferase (ALT), alkaline phosphatase (AP), gamma glutamyl transpeptidase (GGT), serum albumin, prothrombin time (PT), and international normalized ratio (INR)

**Renal function tests: Serum creatinine and blood urea nitrogen with calculation of estimated glomerular filtration rate (eGFR), serum electrolytes (serum sodium and potassium)

***12-lead ECG: if any reported abnormality, 12-lead ECG was repeated every week until improvement or completing the drug course

****24-h Holter ECG: if baseline was abnormal, follow-up 24-h Holter ECG was done at week 4 of treatment

*CBC*, complete blood count; *HBV*, hepatitis B virus; *HCV*, hepatitis C virus; *RNA*, ribonucleic acid

#### Study outcomes

HCV viral load was assessed at week 4 of treatment to detect rapid virological response (RVR), at the end of treatment (week 12) to detect end of treatment response (ETR), and at week 24 from the start of treatment to detect sustained virological response (SVR12). In addition, end of treatment eGFR was calculated. Adverse events assessment was done at the end of treatment and at week 24. Breakthrough is defined as the detection of viral load at week 12 at the end of treatment after a negative RVR. Relapse is the detection of viral load during the follow-up period in patients who achieved ETR but failed to achieve SVR12 [13].

#### Statistical methods

Data were verified, coded by the researcher, and analyzed using software, Statistical Package for Social Science IBM-SPSS Statistics version 24.0 (IBM-SPSS Inc., Chicago, IL, USA). Quantitative parametric data, such as laboratory tests, eGFR, and ECG findings, were expressed as mean ± standard deviations (SD), while non-parametric data including *Z* scores and HCV RNA values were expressed as median and inter-quartile range (IQR). Qualitative data (sex, clinical data, fibrosis stages, and AE) were expressed as frequency and percentages. Paired comparisons of pre- and post-treatment laboratory values were performed using the paired *t*-test for normally distributed data and the Wilcoxon signed-rank test for skewed data. A *p*-value of less than 0.05 was considered significant.

## Results

### Basic demographic data

**Fig. 1:**
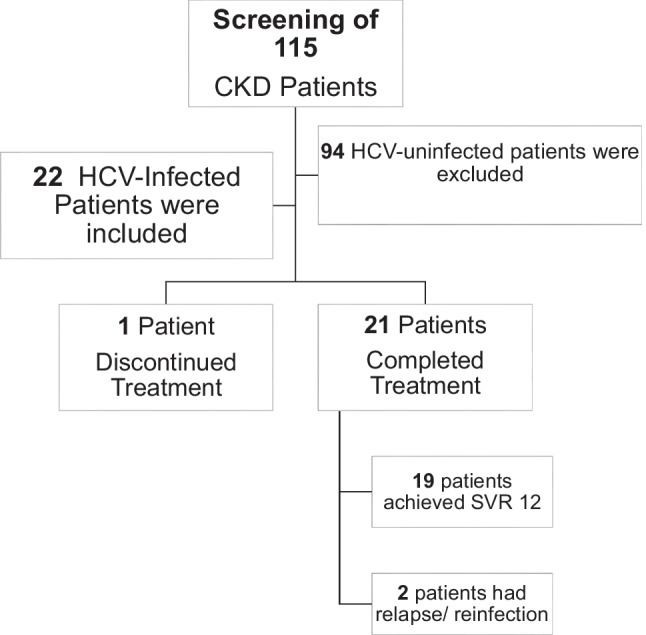
Screening and management of HCV among CKD patients in Pediatric Nephrology Unit, Cairo University Pediatric Hospital, Faculty of Medicine, Cairo University from May 2021 to September 2024. CKD, chronic kidney disease; HCV, hepatitis C virus

From May 2021 to September 2024, 115 CKD pediatric patients on maintenance HD attending the Pediatric Nephrology Unit, Cairo University Pediatric Hospital, Kasr AlAiny School of Medicine, Cairo University, were screened for HCV RNA by PCR. Twenty-two patients tested positive and were included in the study (Fig. 1). They were 14 females (63.6%) and 8 males. Their age ranged from 7.5 to 15.9 years with a median (IQR) of 12 (9.25–13.5) years. Thirteen patients (59%) were born to consanguineous parents (Table 2). They were diagnosed with CKD stage 5 and scheduled for regular HD at a median (IQR) age of 6 (3.5–9) years (range 2–12 years). Their median (IQR) age at first detection of HCV was 8 (7–10) years, with a range of 2.5–13.5 years. All patients had multiple risk factors for acquisition of HCV infection apart from the HD process. Of note, all patients had a history of blood product transfusions, hospitalization, cannulation, and arterio-venous shunt operations. One patient had maternal HCV, and three others (13.6%) had other HCV-positive family members.

**Table 2:** The demographic criteria of the studied cohort

Variable	Results	Notes
Primary renal disease	Ciliopathies: 5 patients Congenital anomalies of kidney and urinary tract: 4 patients Steroid resistant nephrotic syndrome: 3 patients Unrevealed primary renal disease: 10 patients (45.5%)	
Surgical operations	11 patients	Nephrectomy, splenectomy, cholecystectomy, ureteric implantation, hydrocele repair and orthopedic surgery
Dry weight (mean)	26.08 ± 9.5 kg	Range: 17–58 kg
Weight *Z* scores, frequency (percent)	Normal: 3 (13.5%) Underweight: 19 (86.4%)	Underweight Mild: 3 (13.6%) Moderate: 8 (36.4%) Severe: 8 (36.4%)
Height (mean)	120 ± 12.4 cm	Range: 103–152 cm
Height *Z* scores, median (IQR)	−3.9 (−4.7 to −2.57)	Stunted (100%) Mild: 3 (13.6%) Moderate: 3 (13.6%) Severe: 16 (72.8%)
Hepatomegaly	10 patients	Mean size below costal margin At MCL: 6.2 ± 2.8 cm (range: 2–11 cm) At mid-line: 5 ± 2.5 cm (range: 2–9 cm)
Splenomegaly	5 patients	Mean size subcostal 7.2 ± 4.3 cm (range: 3–15 cm)
FibroScan	F0: 12 patients F1: 4 patients F2: 2 patients F4: 4 patients	

*MCL*, mid clavicular line

### Clinical assessment

Before HCV treatment, all patients in the study group were examined by an experienced pediatric hepatologist. None of them exhibited jaundice, palmar erythema, spider nevi, fetor hepaticus, hepatic encephalopathy, purpura, abdominal rigidity, tenderness, or superficial masses. One patient had scratch marks, another had joint deformity, and another had generalized edema. Four patients had abdominal distension; one of them had ascites. One patient had dilated veins on the abdominal wall, and another had an umbilical hernia. Additionally, ten patients had scars on the abdominal wall, including post-peritoneal dialysis, nephrectomy, and splenectomy, one each. The latter had ciliopathy with congenital hepatic fibrosis and portal hypertension. The liver was palpable in 10 patients (Table 2), three had rounded edges, and seven had sharp edges. Eight livers were firm in consistency, while two were soft. The eGFR was below 15 mL/min/1.73 m^2^ in all but three whose eGFR was between 32.7 and 38.3 mL/min/1.73 m^2^; mean basal eGFR for the whole cohort was 11.9 ± 8 mL/min/1.73 m^2^. All patients had complex drug prescriptions; however, none had any DDI with SOF/LDV. None of them had experienced previous HCV treatment.

### Cardiological assessment

Cardiological screening revealed that 17 patients had normal basal ECG and 24-h Holter ECG, while four patients had sinus tachycardia, and one patient had ST segment depression. Their echocardiographic assessment showed that nine patients had left ventricular dilatation, one patient had concentric hypertrophy with systolic dysfunction, one patient had calcified aortic valve leaflets, and the rest had unremarkable echocardiographic findings. Their fractional shortening ranged from 21 to 40% with a mean of 31.9 ± 4.4%.

### Laboratory tests

All patients tested negative for hepatitis B surface antigen and core antibody during virology screening. They all received the hepatitis B vaccine; one patient needed a booster dose of hepatitis B vaccine to enhance his antibody titer to the protective level. The median antibody level of HBsAb was 117.35 mIU/mL (IQR 43.9–844.25 mIU/mL). Most of them had a normal basal profile of liver functions except for ALT, which was elevated above the upper limit of normal in six patients (27%) with mean ± SD (27.1 ± 17.5 IU/L), and AST, which was elevated in five patients (22.7%) (Table 3). The median (IQR) of baseline hepatitis C RNA PCR was 62,446 (6875–604,000) IU/L.

**Table 3:** The baseline and follow-up laboratory investigations of the studied cohort

	Baseline Mean (*N* = 22)	Week 12 Mean (*N* = 21)	Week 24 Mean (*N* = 21)	Δ Basal–week 12 (95% CI/median Δ, *p*)	Δ Week 12–week 24 (95% CI/median Δ, *p*)	Δ Basal–week 24 (95% CI/median Δ, *p*)
Hepatitis C RNA* PCR (IU/mL)	1,180,000	100% undetectable	Two patients detectable	Decreased to undetectable, *p* < 0.0001	2 patients relapsed, *p > *0.05	Decreased overall, 2 relapses, *p* < 0.001
Hemoglobin (g/dL)	12.3	12.5	12.6	− 0.2 [− 0.4 to 0.0], *p* = 0.08	− 0.1 [− 0.3 to 0.1], *p* = 0.22	− 0.3 [− 0.6 to 0.0], *p* = 0.07
Total bilirubin** (mg/dL)	1.08	0.86	0.72	Median Δ = 0.22, *p* < 0.0001	Median Δ = 0.14, *p* < 0.0001	Median Δ = 0.36, *p* < 0.0001
ALT (IU/L)	46.2	28.7	22.9	17.5 [14.6–20.4], *p* < 0.0001	5.8 [3.9–7.7], *p* < 0.0001	23.3 [20.1–26.5], *p* < 0.0001
AST (IU/L)	39.4	24.6	19.8	14.8 [12.3–17.3], *p* < 0.0001	4.8 [3.1–6.5], *p* < 0.0001	19.6 [17.0–22.2], *p* < 0.0001
GGT** (IU/L)	91.2	66.4	53.5	Median Δ = 25.0, *p* < 0.0001	Median Δ = 13.1, *p* < 0.0001	Median Δ = 38.1, *p* < 0.0001
AP (IU/L)	295.6	258.2	221.7	37.4 [31.2–43.6], *p* < 0.0001	36.5 [30.0–43.0], *p* < 0.0001	73.9 [63.1–84.7], *p* < 0.0001
Albumin (g/dL)	3.91	4.06	4.14	− 0.15 [− 0.21 to − 0.09], *p* < 0.001	− 0.08 [− 0.14 to − 0.02], *p* = 0.01	− 0.23 [− 0.30 to − 0.16], *p* < 0.0001
PT-INR	1.13	1.07	1.04	0.06 [0.03–0.09], *p* < 0.001	0.03 [0.01–0.05], *p* = 0.002	0.09 [0.06–0.12], *p* < 0.0001

Paired* t*-test was used

*Cochran’s *Q *test

**Wilcoxon signed-rank test

*AP*, alkaline phosphatase; *ALT*, alanine aminotransferase; *AST*, aspartate aminotransferase; *CI*, confidence interval; *GGT*, gamma glutamyl transpeptidase; *PCR*, polymerase chain reaction; *PT*-*INR*, prothrombin time-international normalized ratio; *RNA*, ribonucleic acid

### Drug administration and safety concerns

According to their body weight, 20 patients (90.9%) received half tablets (sofosbuvir 45 mg/ledipasvir 200 mg), while two patients weighing > 35 kg received the whole tablet (sofosbuvir 400 mg/ledipasvir 90 mg) as a single oral daily dose for a 12-week course as previously described. Only one patient experienced vomiting severe enough to discontinue the medication during his first week of treatment (CTCAE grade 3). The remaining 21 patients completed the treatment course; nine patients (42.8%) reported mild AE (CTCAE grade 1) as illustrated in Table 4. No SAEs were reported during the study. The follow-up ECG recordings during treatment and at the end of treatment, as well as the end of treatment 24-h Holter, recorded no serious cardiac AE. Two patients developed prolonged QT intervals in their on-treatment ECG recordings that necessitated close monitoring by an experienced pediatric cardiologist with no need to interrupt their course of treatment. Of note, there were no recorded remarkable changes in their laboratory tests (Table 3).

**Table 4:** The reported adverse events for sofosbuvir/ledipasvir by the study population

	Baseline *N* = 22 *N* (%)	Week 4 *N* = 21 *N* (%)	Week 12 *N* = 21 *N* (%)	Week 24 *N* = 21 *N* (%)	CTCAE grade	*P*-value
Jaundice	0	0	0	0	-	
Skin rash	0	0	0	0	-	
Asthenia	4 (18.2)	2 (9.5)	2 (9.5)	2 (9.5)	Grade 1	0.99
Fatigue	4 (18.2)	2 (9.5)	3 (14.2)	2 (9.5)	Grade 1	0.99
CNS manifestations						
Headache Dizziness Insomnia Irritability	6 (27.3) 4 (18.2) 2 (9.1) 8 (36.4)	5 (23.8) 3 (14.2) 1 (4.8) 4 (19)	3 (14.2) 0 1 (4.8) 3 (14.2)	3 (14.2) 0 0 1 (4.8)	Grade 1 Grade 1 Grade 1 Grade 1	0.98
Respiratory manifestations						
Cough Dyspnea	5 (22.7) 4 (18.2)	2 (9.5) 0	2 (9.5) 1 (4.8)	1 (4.8) 1 (4.8)	Grade 1 Grade 1	0.94 0.76
GIT manifestations						
Nausea Vomiting Diarrhea	1 (4.5) 0 1 (4.5)	1 (4.8) 1 (4.8) 2 (9.5)	0 0 1 (4.8)	1 (4.8) 0 1 (4.8)	Grade 1 Grade 3 Grade 1	0.97 0.98 0.95
Heart rate (beats/min)						
Mean ± SD	89.8 ± 20.5	94.9 ± 14.2	90.4 ± 16.6	94.5 ± 12.7	-	0.88

*CNS*, central nervous system; *CTCAE*, Common Terminology Criteria for Adverse Events; *GIT*, gastrointestinal tract; *SD*, standard deviation

### Efficacy assessment

The RVR at week 4 of treatment, as well as at the ETR, for the 21 patients who completed their treatment course showed clearance of HCV RNA in all patients (100%). However, SVR12 showed that two patients had a positive HCV PCR (9.5%), indicating relapse versus re-infection. These two patients were 8 and 14 years old; the former had F1 on FibroScan assessment and baseline ALT 64 IU/L, while the latter had F0 and baseline ALT 20 IU/L. Unfortunately, both died with pulmonary edema secondary to their kidney failure 5 and 6 months after the end of treatment. Nineteen out of 22 patients (86.4%) achieved SVR12 with clearance of HCV infection.

## Discussion

The KDIGO guideline 2022 update has recommended HCV management in patients with CKD [3]. The recently developed DAAs have enabled a more effective and tolerable approach of HCV management in all patients with CKD, including those on maintenance HD therapy and those post kidney transplantation [5]. This contrasts with the previously used approach of therapy with IFN with or without ribavirin that had low cure rates with multiple side effects and discontinuation of therapy [14, 15]. In a study that has been conducted in our center for evaluation of interferon monotherapy in 17 patients with CKD on maintenance HD therapy, although they achieved a relatively high ETR (76.5%), SVR could not be assessed because of the high dropout rate (88%). Furthermore, patients experienced frequent multiple drug AEs mainly in the form of constitutional and hematological side effects with a discontinuation rate of 11.8% [16].

The current study included 22 patients with CKD on maintenance HD with HCV infection, proven by nucleic acid testing, as recommended in screening of HCV in dialysis units [3]. They were all asymptomatic, and only six patients had elevated baseline ALT confirming the need for use of nucleic acid testing in HCV screening of such a high-risk population. Elevated ALT level was found to be sensitive and specific for acute HCV infection among HD patients but with inadequate positive predictive value [17]. Although ALT was elevated in only 27% of the present study, the significant decline of its levels with HCV treatment (*p*-value < 0.0001) may validate the suggestion of baseline and monthly check of ALT levels in HD patients in the KDIGO guideline 2022 update [3].

The NS5B inhibitor SOF is a significant drug in multiple DAA regimens. Its renal metabolism and elimination posed a concern regarding its use in CKD patients [18]. SOF has been FDA approved for use in CKD patients on dialysis after many studies that revealed its high efficacy, minimal AE, and stable eGFR and creatinine levels in addition to non-accumulating levels of SOF and its metabolites in between dialysis sessions among this targeted population [19, 20]. SOF is not recommended as monotherapy because of its lower efficacy; on the other side, SOF-based regimens are thoroughly evaluated and authorized for all CKD patients including those on HD [3]. In the present study, we reported 100% RVR and ETR responses, and 90.5% SVR12 in 19 out of 21 patients who completed the treatment course. This is comparable to SVR12 recorded in many related studies evaluating SOF/LDV in adults with CKD on HD: 94% [21], 80% [22], and from 94 to 97% in a systematic review [5]. It is noteworthy that this reported SVR12 is inferior to the reported SVR12 in non-CKD pediatric patients in our center in a previous study, which was 100% [23]. Two patients (9.5%) among this study cohort tested positive for HCV RNA by PCR at week 12 after the end of treatment, which could be considered a relapse or re-infection. There is reported evidence that HCV relapse is not uncommon among patients on HD in Egypt [24, 25] with multiple incriminated risk factors; a sofosbuvir-based regimen is considered one of these risk factors [24]. Besides, despite improving standards of infection control in HD centers in Egypt, there are some recorded cases of seroconversion while on HD indicating ongoing HCV transmission [26].

Considering SOF/LDV safety, we recorded high tolerance in all patients with minimal AE. Only one patient discontinued the drug because of persistent vomiting. Furthermore, there were no recorded incidents of transient elevation of transaminases or hemoglobin drop or worsening of kidney functions during treatment. This is close to its tolerance in adults. In three prospective studies, no SAEs were reported [21, 27, 28]. Also, in a large-scale multicenter cohort study in Egypt among adult chronic HCV Egyptian patients with CKD, SOF-based regimens were found to be safe with a 0.8% discontinuation rate and SAE reported in 0.1% of cases [22]. The most reported AEs in adults were headache, fatigue, and nausea [18, 19]. These manifestations were strongly related to renal impairment as they were recorded in the basal survey of our pediatric patients. Furthermore, there was no reported worsening of these complaints in our cohort. Although we noted prolonged QT intervals in initial on-treatment ECG records in two of our patients, there was no need for drug discontinuation with consecutive improvement in follow-up ECG. SOF/LDV was found to have no cardiovascular AEs in HCV-infected children in a former study in our center [29]. Saxena et al. [19] reported cardiac SAEs in 4% of their study group of SOF-based regimen in adults with renal impairment with no mentioned details about the need for drug cessation.

One of the limitations of this study is that we did not investigate in depth the degree of cirrhosis in our patients and its implication on response to treatment and adverse effects. However, HCV is not expected to cause cirrhosis that early in children and adolescents. Among our four patients who had F4 on FibroScan assessment, two had congenital hepatic fibrosis, while the other two had congestive hepatomegaly by ultrasound. In addition, further studies with longer observation periods are recommended for profound safety assessment of SOF/LDV in such high-risk groups.

In conclusion, to our knowledge, this is the first study to investigate SOF-based treatment of chronic HCV pediatric patients with CKD on HD. SVR12 was relatively inferior to HCV-infected children without CKD (86.4% vs. 100%). One patient (4.5%) experienced remarkable AE (CTCAE grade 3) in the form of persistent vomiting necessitating discontinuation of the drug. SOF/LDV was found to be effective with SVR12 of 86.4% in HCV-infected CKD patients on maintenance HD.

## Supplementary Information

Below is the link to the electronic supplementary material.

(PPTX 1.86 MB)

## Data Availability

The datasets generated during and/or analyzed during the current study are available from the corresponding author on reasonable request.

## Abbreviations

AE: Adverse event
CKD: Chronic kidney disease
CTCAE: Common Terminology Criteria for Adverse Events
DAA: Direct acting antiviral
DDI: Drug–drug interactions
eGFR: Estimated glomerular filtration rate
ETR: End of treatment response
HCV: Hepatitis C virus
HD: Hemodialysis
IQR: Interquartile range
PCR: Polymerase chain reaction
RVR: Rapid virological response
SAE: Serious adverse event
SD: Standard deviations
SOF: Sofosbuvir
SOF/LDV: Sofosbuvir/ledipasvir
SVR: Sustained virological response
