# Supplementary material for: Efficacy and safety of SOF/LDV in HCV-infected children and adolescents on hemodialysis: a prospective single-center observational study
Source: Pediatr Nephrol. 2026 Mar 9;41(8):2583–90. doi: 10.1007/s00467-025-07112-6 (PMC13337618; doi:10.1007/s00467-025-07112-6)
Supplement: Supplementary file 1 — (PPTX 1.86 MB) [file 467_2025_7112_MOESM1_ESM.pptx]

## Slide 1
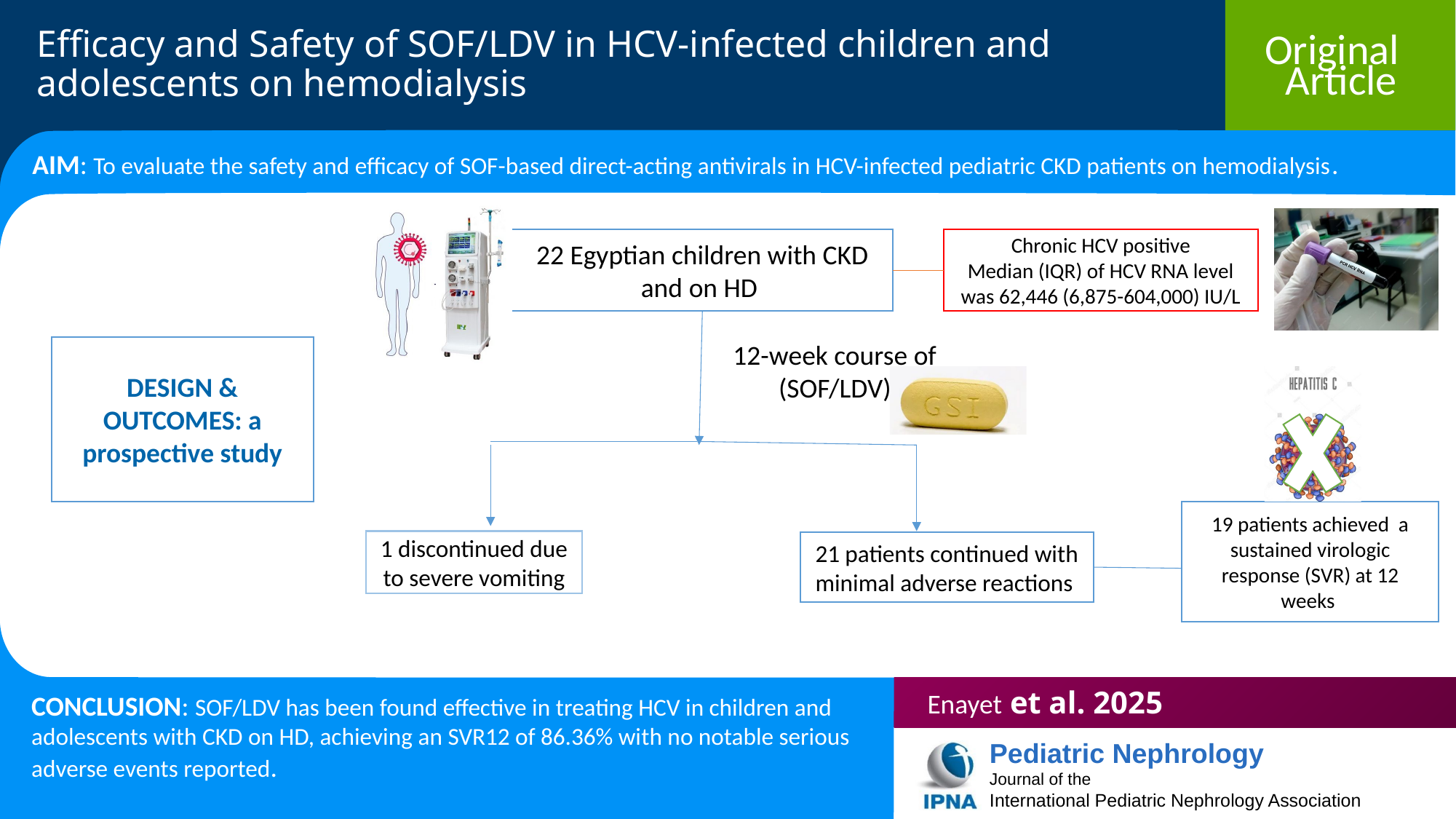

Efficacy and Safety of SOF/LDV in HCV-infected children and adolescents on hemodialysis
AIM: To evaluate the safety and efficacy of SOF-based direct-acting antivirals in HCV-infected pediatric CKD patients on hemodialysis.
22 Egyptian children with CKD and on HD
Chronic HCV positive
Median (IQR) of HCV RNA level was 62,446 (6,875-604,000) IU/L
DESIGN & OUTCOMES: a prospective study
12-week course of (SOF/LDV)
19 patients achieved a sustained virologic response (SVR) at 12 weeks
1 discontinued due to severe vomiting
21 patients continued with minimal adverse reactions
Enayet et al. 2025
CONCLUSION: SOF/LDV has been found effective in treating HCV in children and adolescents with CKD on HD, achieving an SVR12 of 86.36% with no notable serious adverse events reported.
